# Peak Nasal Inspiratory Flow Evaluation as an objective method of measuring nasal airflow

**DOI:** 10.1590/S1808-86942011000400011

**Published:** 2015-10-19

**Authors:** Rodrigo Ubiratan Franco Teixeira, Carlos Eduardo Monteiro Zappelini, Fábio Silva Alves, Everardo Andrade da Costa

**Affiliations:** 1 MSC in Collective Health - FCM/UNICAMP; 2 Resident Physician - Department of Otorhinolaryngology - Santa Casa de Campinas; 3 Resident Physician - Department of Otorhinolaryngology - Santa Casa de Campinas; 4 PhD; Professor - Graduate Program on Collective Health and Otorhinolaryngology, Head and Neck Surgery - FCM/UNICAMP - Thesis Adviser

**Keywords:** nasal obstruction, rapid evaluation, rhinitis

## Abstract

**Abstract:**

Acoustic rhinometry, rhinomanometry and Inspiratory Peak Nasal Flow (IPNF) are used in order to objectively assess nasal patency. These are expensive not very practical tests, except for IPNF, which is a fast, simple and low cost method.

**Objective:**

To assess IPNF in healthy individuals complaining of nose obstruction caused by allergic rhinitis.

**Method:**

IPNF use in 78 individuals with and without rhinitis symptoms.

**Study design:**

Contemporary cross-sectional cohort.

**Results:**

IPNF showed significant results for nasal obstruction, rhinorrhea, pruritus, sneezes and tearing (*p* < 0.001). There was no correlation between the presence of nasal septum deviation and IPNF (*p* = 0.561). We found a positive correlation between IPNF and the Visual Analogue Scale (VAS) for nasal obstruction (*p* = 0.002). In the multiple linear regression model, there was a statistical significance between the values found in IPNF with allergic rhinitis and age (*p* = 0.005 and *p* = 0.023 respectively).

**Conclusion:**

IPNF proved to be a reliable method to detect changes in nasal patency, by obstructive causes as well as inflammatory causes, with an acceptable level of statistical significance, simple, easy to handle, inexpensive and reproducible.

## INTRODUCTION

Nasal breathing is predominant in the human race, from birth all the way to adulthood. Mouth breathing or oral-nasal breathing is physiologically used in cases of higher demand, such as in the practice of physical exercises. More than half of the resistance against airflow in the respiratory tract can be found in the nose. The nasal valve is the region between the nasal septum, the anterior portion of the inferior turbinate and the lower border of the inferior lateral cartilage. It is the main mechanism regulating nasal airflow^1,^^2^.

Besides respiratory well-being, other functions such as swallowing, sleep quality, olfaction, paranasal sinus aeration and middle ear health, among others, depend on proper nasal functioning. Therefore, the development of objective assessment techniques of the nasal function is fundamental to diagnose deviations from normal and proper patient follow up.

Ideally, objective tests which assess nasal patency must be confortable, accurate, standardized and easy to perform, clinically applicable and must not impact nasal anatomo-physiology. Moreover, its reproducibility is fundamental, which is the test's capacity to produce consistent results when independently repeated^3^. The most commonly used objective methods to study nasal airflow are computerized rhinomanometry, acoustic rhinometry and Nasal Inspiratory Peak Flow (NIPF)^4^. The first assesses the air flow in its entire extension in the nasal cavity, the second measures the cross-sectional areas in predetermined points in the nasal cavity^5^ and NIPF, as the very name says, measures the nasal inspiratory peak flow^6^.

NIPF is inexpensive, easily applied, fast, portable, simple to measure, does not depend on computers to analyze the data and has good reproducibility. Both rhinometry as well as the NIPF are accurate to detect nasal obstructive changes, with 0.77 vs. 0.66 of sensitivity, respectively. The methods' specificity is 0.8 with a diagnostic accuracy around 0.7^5^,^7^. The cutting value for the peak flow measured by the NIPF, according to some authors, is less than 120 l/min, with a difference of approximately 35% before and after the use of a nasal vasoconstriction agent^8^. Notwithstanding, these findings are associated with the results from foreign studies, and there are no Brazilian studies which corroborate for a standardization of values found in the NIPF associated with the Brazilian Population. The latter, with 180 million inhabitants, is highly mixed, with the following ethnic composition: whites (49.4%), browns (42.3%), blacks (7.4%), yellow (0.5%) and Indians (0.3%)^10^, each one with its own particular nasal anatomical characteristics, with statistical differences in relation to feeding habits, socioeconomical characteristics, climate and dressing habits.

The present paper aims at assessing NIPF as a method to measure nasal airflow changes and compare the values found upon NIPF in individuals with nasal obstruction by allergic rhinitis and normal controls.

## MATERIALS AND METHODS

The present paper represents a contemporary, cross-sectional cohort study, in which the variable studied is the NIPF behavior in a population of healthy individuals and individuals with rhinitis. The individuals voluntarily participated in the study, and they were selected in a non-randomized fashion. This study was approved by the Ethics and Research Committee under protocol number 824/2009. The study started after the participants signed two copies of the informed consent form, which were also signed by the examining physician.

**Figure 1:**
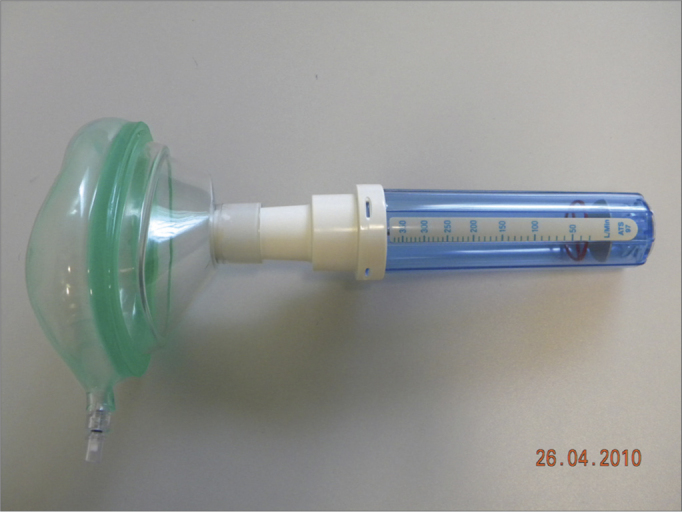
Nasal Inspiratory Peak Flow (NIPF).

**Figure 2:**
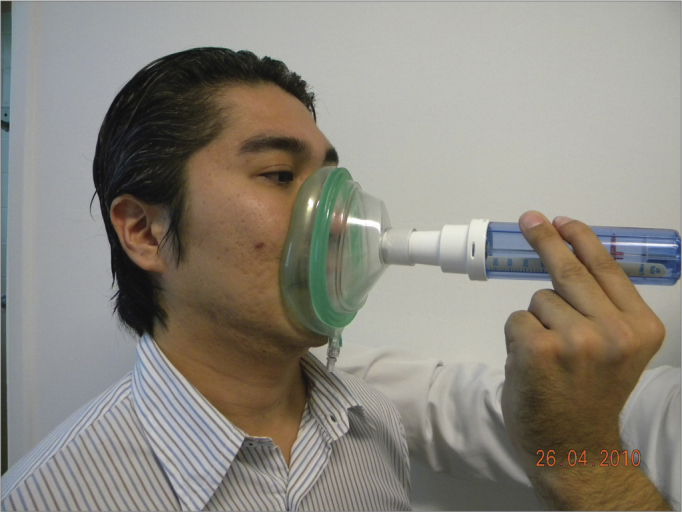
NIPF application.

The sample counted on 78 volunteers with ages between 19 and 67 years, encompassing patients, patient companions, physicians, hospital and clinic employees and other administrative professionals from the institution. All the volunteers were examined by the same ENT physician by means of an interview, physical exam (anterior rhinoscopy and nasal fiber optic exam), and the patients answered the standardized signs and symptoms questionnaire. At this point, rhinitis was diagnosed according to the data collected (interview, physical exam and nasal fibroscopy)^9^, ^10^, ^11^; and then, the participants were broken down into two groups: those with rhinitis and those without it. After that, NIPF was employed in three consecutive individual takes in order to obtain the highest measure. Exclusion criteria were: individuals with upper airway infections on the day of the exam or in the 14 days prior to it; participants who chronically used nasal decongestants, anti-histamine, anticholinergic or steroid (topic nasal or systemic) agent; nasal cavity malformation; nasal polyps or nasal masses, or those who drank alcohol or smoked during the exam. Upon physical exam, the following items were valued: nasal conchae hypertrophy, rhinorrhea, nasal septum deviations, mucosal aspects (nasal conchae hypertrophy, rhinorrhea, nasal septum deviations, mucosal aspects (color, impregnations, signs of bleeding and crust), other changes of the nasal frame and pyramid and the nasal meatuses. Smokers were included, as well as those with nasal septum deviation. All participants were required to report on the use of any kind of drug prior to the assessment. The examinations were carried out in the outpatient wards of the institutions, where there already are facilities for it - proper room, proper chair, table, air conditioning, maintaining room temperature between 22**°**C-24**°**C. The material used to disinfect the nasal fibroscope and NIPF were chlorhexidine, 70% alcohol, hydrogen peroxide and soap. We used an Olympus ENF type P4 fibroscope and a Clement Clark International Limited model IN-CHECK ORAL ATM device to analyze NIPF. This device is made up of a 20cm plastic cylinder, with a diameter varying between 3 and 4 cm, with two ends: one of them has holes from where the inspired air passes, the other end is coupled to a facial mask which is in contact with the volunteer's face. Inside the cylinder there is a diaphragm which moves according to the maximum air inflow. The diaphragm stops moving when flow stops, then measurement is carried out in a scale which varied between 30-370 liters/minute, which is branded on the cylinder's surface (Figures 1 and 2).

**Figure 3:**
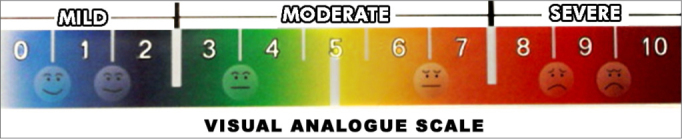
Visual-analogue scale used for nasal obstruction

The devices were disinfected immediately after use. The measures were carried out with the following specifications: 1) Acclimatization - participant seating for 20 minutes in the test area. During this period, the questionnaire was filled out and the physical exam was made; 2) The participant filled out the visual-analogue scale (VAS) concerning nasal obstruction (Figure 3); 3) The patient's NIPF was measured three consecutive times with a 1 minute interval between them^4,^^6^. The exam technique was based on measuring the participant's nasal inspiratory flow when standing up, with the device coupled to the nose's anterior region through a small mask connected to a plastic cylinder through which the forced inspired air passes. The result is seen immediately, similarly to the well-known expiratory peak flow routinely used in pneumology to study the pulmonary expiratory capacity.

The data was descriptively analyzed per absolute (n) and relative(%) frequencies of the categorical variables. For continuous variables we used the mean, standard deviation, median, first and third quartile, maximum and minimum values.

The Student's t and variance analysis (ANOVA) tests were employed in order to compare the NIPF according to demographic and clinical characteristics. The Spearman linear correlation coefficient was used in order to analyze the correlation between NIPF and VAS and age of the subjects in the sample. A multiple linear regression model was adjusted in order to analyze the impact of some clinical and demographic characteristics on the NIPF. We included in the model those variables which had a *p* <0.25 upon multivariate analysis.

The SAS 9.1.3 software (SAS Institute Inc., Cary, NC, USA, 2002-2003) was utilized for data analysis purposes and a statistical significance level of 5% was used.

## RESULTS

Table 1a depicts the clinical and demographic characteristics of this sample with 78 individuals, mostly females - n = 47 (60.3%) in relation to males n=31 (39.7%). We also noticed that 80.8% of the individuals were whites, 95% had not had upper airway infections in the past 14 days, and only 2.7% had had prior nasal or palate surgery. In our sample, 65 individuals (83.3%) did not smoke or had stopped for more than 5 years.

**Table 1a:** Sample clinical and demographic characteristics.

Characteristics	n	%
Gender		
M	31	39.7
F	47	60.3
Ethnics		
White	63	80.8
Black	6	7.7
Other	9	11.5
Smoking		
No	65	83.3
Yes	13	16.7
Nasal obstruction		
No	36	46.2
Yes	42	53.8
Nasal septum deviation		
No	58	74.4
Yes	20	25.6
Inferior turbinate hypertrophy		
0	10	12.8
1	14	17.9
2	31	39.7
3	23	29.5
Nasal secretion		
No	67	85.9
Yes	11	14.1
Rhinitis		
No	43	55.1
Yes	35	44.9

(n=78)

As far as rhinitis symptoms go, 36 participants had nasal obstruction (46.2%). No rhinorrhea, pruritus and sneezing were seen in 79.5%, 65.4% and 83.3%, respectively. This represents a sample of individuals without a predominance of these cardinal symptoms for diagnosing rhinitis; thus, most are normal (55.1%). We have noticed that 74% of the individuals did not have nasal septum deviation and 14.1% had nasal secretion upon nasal-fibroscopy.

Table 1b depicts a mean age of 36.8 years, with ages varying between 19 and 67 years. As to the Visual Analogue Scale for nasal obstruction, the mean value found was 3.7, which score varies between 0 and 10.

**Table 1b:** Sample demographic and clinical characteristics.

	AGE	Visual-analogue scale for nasal obstruction
n	78	78
Mean	36,8	3,7
Standard deviation	12,2	2,9
Median	33	3,5
1^st^ quartile	27	1
3^rd^ quartile	48	6
Minimum	19	0
Maximum	67	10

(n=78)

Table 2 depicts the chronic use of nasal decongestants and anti-histamine agents was only prevalent in rhinitis patients, in 14.3%, which was not found in those without rhinitis, with a p-value of *p*=0.008. Of the participants who reported having been exposed to inhaling chemical products, 25.7% had rhinitis and 11.6% did not, with a p-value of p=0.049. Nasal obstruction was found in 85.7% of the patients with rhinitis and in 27.9% of those without it (*p*<0.001). Nasal pruritus was also prevalent among rhinitis patients, with 65.7%, compared to the 9.3% found among those without it (*p*<0.001). Rhinorrhea was seen in 42.9% of those with rhinitis and in 2.3% of those without it (*p*<0.001). Sneezing and tearing were also more prevalent among rhinitis patients, with 68.6% and 34.3% compared to 7% and 2,3% of those who do not have it, respectively, both with a p-value of *p*<0.001. Nasal secretion noticed by nasal fibroscopy was seen in 28.6% of the rhinitis patients and in 2.3% of those without it (*p*<0.001). The variables: nasal septum, mold, moisture, smoking, a pet in the house, were not statistically significant in the linear regression model.

**Table 2:** Clinical characteristics, according to the presence of rhinitis.

	Rhinitis				
Clinical characteristics	Yes		No		*p* Value
	n	%	n	%	
Smoking					0.099¢
No	32	91.4	33	76.7	
Yes	3	8.6	10	23.3	
Exposure to inhaling chemical products					0.049¢
No	26	74.3	38	88.4	
Yes	9	25.7	5	11.6	
Home exposure to mold					0.581¢
No	29	82.9	38	88.4	
Yes	6	17.1	5	11.6	
Pet					0.065¢
No	16	45.7	29	69.0	
Yes	19	54.3	14	31.0	
Moisture					0.988¢
No	29	82.9	35	81.4	
Yes	6	17.1	8	18.6	
Nasal obstruction					<0.001¢
No	5	14.3	31	72.1	
Yes	30	85.7	12	27.9	
Rhinorrhea					<0.001¢
No	20	57.1	42	97.7	
Yes	15	42.9	1	2.3	
Pruritus					<0.001¢
No	12	34.3	39	90.7	
Yes	23	65.7	4	9.3	
Sneezing					<0.001¢
No	11	31.4	40	93.0	
Yes	24	68.6	3	7.0	
Tearing					<0.001¢
No	23	65.7	42	97.7	
Yes	12	34.3	1	2.3	
Nasal septum deviation					0.561¢
No	25	71.4	33	76.7	
Yes	10	28.6	10	23.3	
Nasal secretion (rhinoscopy)					0.011£
No	27	77.1	42	97.7	
Yes	8	22.9	1	2.3	
Nasal secretion (nasal fibroscopy)					0.002¢
No	25	71.4	42	97.7	
Yes	10	28.6	1	2.3	

£ Fisher's exact test. (n=78).

¢ Pearson's chi-square test.

On Table 3, the mean NIPF values for healthy patients who reported no obstruction was 151.4 l/min and 123.6 l/min for those who reported having nasal obstruction (*p*=0.002). As far as nasal conchae hypertrophy (CH) is concerned, by means of the nasal endoscopic exam, the mean NIPF value found for grade 0 CH (no hypertrophy) was 163.0 l/min; for grade I CH, the mean value was 141.2 l/min; for grade II CH, the mean value found was 138.2 l/min, and for grade III CH, the mean value found was 116.4 l/min. In this regression, the p value was 0.008.

**Table 3:** NIPF comparison according to sample demographic and clinical characteristics.

Characteristics	n	Mean	Standard deviation	Median	1^st^ quartile	3^rd^ quartile	Minimum	Maximum	*p*-value
Gender									0,631¥
F	47	134.7	43.0	130	100	150	50	250	
M	31	139.0	31.8	140	120	155	50	200	
Ethnicity									0.999£
White	63	136.3	41.8	130	110	150	50	250	
Black	6	136.7	28.0	140	110	160	100	170	
Other	9	136.7	20.6	150	130	150	100	160	
Rhinitis									<0.001¥
No	43	154.3	37.7	150	125	170	90	250	
Yes	35	114.0	27.4	115	100	130	50	160	
Inferior nasal turbinate hypertrophye									0.008£
0	11	163.0a	42.3	150	150	200	100	250	
1	13	141.2	41.0	130	110	170	100	240	
2	33	138.2a.b	40.8	150	120	155	50	220	
3	21	116.4a.b	19.4	110	100	130	80	150	
Nasal obstruction									0.002¥
No	36	151.4	42.5	150	115	180	60	250	
Yes	42	123.6	30.3	127.5	100	150	50	200	
Nasal Septum deviation									0.878¥
No	58	136.8	39.0	130	110	150	50	250	
Yes	20	135.3	39.1	140	100	150	50	200	

e Equal symbols have statistically significant differences (*p*<0.05), according to Bonferroni's test.

¥ Student's t-test (n=78).

£ Variance analysis test (ANOVA).

The mean NIPF value for rhinitis patients was 114,0 l/min, and for those without it, the mean value found was 154.3 l/min, with a p value < 0.001.

As to the nasal obstruction complaint, the mean NIPF for those without rhinitis was 151.4 l/min and 123.6 l/min for those with obstruction, with a p value of 0.002. DSN, race and gender were not statistically significant.

Table 4, shows a significant association (*p*=0,.02) between the values found in the visual analogue scale and NIPF. The same was not seen between age and NIPF.

**Table 4:** Spearman correlation coefficient (r) between NIPF with VAS and age.

	r	Valor *p*
Age	-0.15	0.2
VAS	-0.41	<0.001

(n=78)

**Graph 1:**
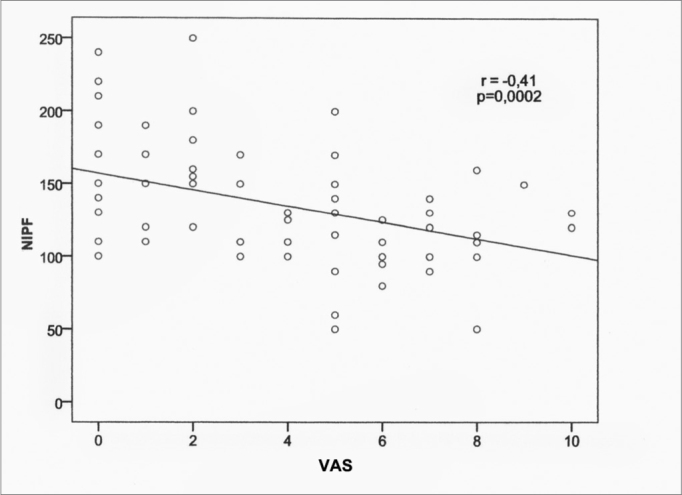
Correlation between the NIPF and the Visual Analogue Scale (n=78).

In the Graph 1, we notice a strong association between NIPF and VAS values, with a *p*<0.001.

Table 5 shows that the age and rhinitis variables were significant for NIPF values in a multiple linear regression model.

**Table 5:** Results from the peak-flow multiple linear regression.

	Coefficient (b)	Standard deviation	t	*p*>t
Intercepto	135.6	15.4	8.82	<0.001
RHINITIS (N/S)	34.7	11.9	2.91	0.005
VAS	-0.5	1.9	-0.27	0.788
Nasal obstruction (N/S)	4.4	9.8	0.45	0.652
Nasal conchae hypertrophy (ref=3)				
0	20.3	13.4	1.51	0.135
1	6.1	12.9	0.48	0.635
2	11.8	9.5	1.24	0.220
Age	-0.7	0.3	-2.32	0.023

Determination coefficient R2=36.7% (n=78).

## DISCUSSION

Statistics show rhinitis prevalence between 30% and 40% in the population^11^, similarly to what is found inthe present paper, which was of 44.9%. In the US alone, there are approximately 40 million people with allergic rhinitis^12^. As far as the nasal septum is concerned, 25% of the individuals had an obstructive deviation. In 2008, authors published in the literature that 75% and 80% of the people have nasal anatomic changes, most of them with nasal septum deviation^13^. Later on, in 2009, other authors showed that statistics concerning nasal septum deviations are different, mostly because of the difficulty in classifying deviations in obstruction shape and intensity^14^.

NIPF, as well as rhinometry, is an important complementary test to support diagnosis of nasal function and structural changes. In the present study, we found rhinitis patients with a mean NIPF value between 114 l/min and 154.3 l/min in healthy individuals. This difference was statistically significant, which corroborates to the use of NIPF in the diagnosis of obstruction. In the literature, authors usually adopt a cutting point of 120 l/min for symptomatic individuals, and sensitivity and specificity higher than 75%^7^.

Mucha et al.^12^ have already used the NIPF as an instrument in the assessment and treatment of individuals with allergic rhinitis, thus showing its usefulness in patients with this disease. Bhatia et al.^15^ also utilized the NIPF as the only assessment method for nasal patency improvement in seasonal rhinitis, comparing the treatment between desloratadine and intranasal budesonide. In the present study, we noticed a significant increase in NIPF values comparing the values before the treatment in both groups (*p*<0.01)^15^.

Lund et al. ^16^ utilized the NIPF, together with the acoustic rhinometry to compare the response to intranasal treatment with fluticasone and beclometasone in the severe nasal polyposis. They found a mean NIPF increase after treatment, from 76 l/min after the symptomatic use of fluticasone and 69 l/min with beclometasone in relation to the basal values, showing a statistically significant difference between the two groups^16^.

McWhorter et al.^17^, studied the role of the soft-palate tensor muscle in upper airway collapse, and observed that after its electrical stimulation, there was a 25% increase in the nasal inspiratory peak flow mean value. This shows that the NIPF is useful to help detect palate deformities.

As we could see, in the numerous papers mentioned, nasal patency checking with NIPF has been well established, in the presence of rhinitis, nasal polyps, in the assessment of treatment, comparing nasal anti-inflammatory treatment modalities and the presence of structural deformities. NIPF is sensitive to detect changes to the nasal conchae size, since these are directly associated with nasal flow resistance. According to some authors, the nasal obstruction pathogenesis is complex, and it depends on three main events: mucosa inflammatory edema, vascular congestion and mucosa hypersecretion^18^. Thus, the same rationale is applicable to the presence of rhinitis. Thus, NIPF can be used in the diagnosis of small changes to the nasal patency, such as, for example, in nasal mucosa provocation tests.

The first publications regarding its use started to appear in the mid 90's^3,^^9^. Notwithstanding, most of the papers are recent and done abroad, which shows that such method is not widely used in Brazil.

In the present study, we noticed that the following variables: nasal obstruction, nasal conchae hypertrophy (CH) and rhinitis were predictive of the values found for by NIPF.

Some authors showed that acoustic rhinitis associated with anterior rhinomanometry was more sensitive than the NIPF to detect changes to the nasal patency, for assessing each nasal cavity individually, which takes into account the nasal cycle and possible septum deviations. The NIPF detects the nasal inspiratory peak flow as the summation of the values obtained from both nasal cavities simultaneously, disregarding the fact that each nasal cavity, separately, with a good applicability to detect changes to the nasal patency as a whole^19^.

Considering Graph 1, there was a linear correlation between the NIPF value and the Visual Analogue Scale (*p*=0.002), in other words, the greater the nasal obstruction reported by the participant, the lower the expected NIPF value. Similar values were obtained in a study in which they reported a strong association between the VAS and the acoustic rhinometry for nasal obstruction (*p* < 0.001), making it useful to predict nasal obstruction. Still, according to the authors, the VAS can be used in clinical practice to quantify the nasal obstruction^20^. We did not find in the literature any correlation between the NIPF and VAS.

Lund and Scadding^21^ did a study analyzing the objective measures of nasal cavity endoscopic surgery, which showed that the NIPF or the rhinomanometry improved after surgery, despite important subjective improvements reported by the patients insofar as the nasal obstruction is concerned. Nonetheless, Marais et al.^22^ showed increases in NIPF after septoplasty, as did Cook et al.^23^ after laser treatment for rhinitis.

The NIPF has been utilized to assess the efficacy of nasal dilating agents, when used to manage nasal wing collapse during inspiratory maneauvers^24^ during intensive exercise^25^ or longstanding^26,^^27^. More recently, Bjornsdottir et al.^28^ examined the effect on individuals allergic to cat hair, and showed that after proper treatment, there was a significant improvement in NIPF values and better symptom scores, when compared to a control group.

And finally, in the multiple regression model, rhinitis (*p*=0.005) and age (*p*=0.023) were significant. As rhinitis was added to the model, variables such as: obstruction, rhinorrhea, nasal conchae hypertrophy, pruritus and tearing, which are cardinal for the diagnosis of rhinitis, lost strength when analyzed alone. Now, as far as age is concerned, the higher it is, the lower the expected value for the NIPF. This is due to a probable anatomical change to the nasal tip and narrowing of the internal nasal valve, usually seen as age increases^2,^^6^.

Therefore, NIPF is one more tool in our existing arsenal, to aid in the diagnosis as well as to follow up on nasal clinical or surgical treatments. It is simple and easy to handle device; not expensive and reproducible.

## CONCLUSION

*Nasal Inspiratory Peak Flow* (NIPF) proved to be a reliable method to detect changes to nasal patency due to obstructive or inflammatory causes. It indicated a pattern of definitive values, with one acceptable statistical significance, for individuals with and without rhinitis.

Nonetheless, further studies are needed in order to totally understand and standardize the use of NIPF.
